# Monocolonization with *Bacteroides thetaiotaomicron* exerts region-specific effects on Alzheimer’s disease-related traits in the murine brain

**DOI:** 10.1128/spectrum.00744-25

**Published:** 2026-01-20

**Authors:** Vu Thu Thuy Nguyen, Svenja König, Henning Formes, Zukaa Al Taleb, Florian Steinert, Bernd Bufe, Simone Eggert, Simone Stegmüller, Yannik Schermer, Elke Richling, Stefan Kins, Christoph Reinhardt, Kristina Endres

**Affiliations:** 1 Department of Psychiatry and Psychotherapy, University Medical Center of the Johannes Gutenberg-University Mainz, Mainz, Germany; 2 Department of Human Biology and Human Genetics, University RPTU Kaiserslautern-Landau, Kaiserslautern, Germany; 3 Center for Thrombosis and Hemostasis (CTH), University Medical Center of the Johannes Gutenberg-University Mainz 684352, Mainz, Germany; 4 Faculty of Computer Sciences and Microsystems Technology, University of Applied Sciences Kaiserslautern 160541 , Zweibrücken, Germany; 5 Department of Neurogenetics, Max Planck Institute for Multidisciplinary Sciences 28282, Göttingen, Germany; 6 Department of Chemistry, Division of Food Chemistry and Toxicology, University RPTU Kaiserslautern-Landau 26562, Kaiserslautern, Germany; 7 German Center for Cardiovascular Diseases (DZHK), Rhein-Main, Germany; The Chinese University of Hong Kong, Hong Kong, Hong Kong

**Keywords:** germ-free animals, gut microbiome, gut-brain-axis, Alzheimer's disease

## Abstract

*Bacteroides thetaiotaomicron* (*B. theta*) dominates the gut microbiome of most mammals. This strictly anaerobic gut symbiont colonizes the mucus layer of host intestinal epithelial cells in both healthy and diseased conditions. Reduced neuronal and vagal afferent innervation observed in germ-free mice was found to be normalized by colonization with *B. theta*. In addition to deficits in gut innervation, germ-free mice have been reported to have reduced neuronal number and neurotransmitter levels in the brain. Here, we investigated the hallmarks of Alzheimer’s disease (AD) in the brain of germ-free mice compared to mice mono-colonized with *B. theta*. We analyzed the number of mature neurons, neurotransmitter transporters, amyloid precursor protein processing, and inflammatory status in three brain regions: the hippocampus, prefrontal cortex (PFC), and cerebellum. The hippocampus and the PFC are regions thought to be highly susceptible to pathogenesis, whereas the cerebellum is thought to be only mildly affected. Interestingly, secretion of neuroprotective sAPPα decreased in hippocampus and remained unchanged in PFC, while levels were increased in the cerebellum in response to bacterial colonization. In addition, the number of presynaptic boutons increased in the hippocampus but remained unaffected in the cerebellum.

**IMPORTANCE:**

The gut microbiome has been reported to not only contribute to diseases of the gastrointestinal tract but also to interfere with and potentially even initiate diseases of other organ systems, such as the brain. Interference with the gut microbiome has been shown to elicit cognitive changes, for example, in rodent models of AD. Colonization with the common gut microbe *B. theta* not only affected the brain *per se* in our study but also showed specific brain region-dependent effects related to AD. This implies that evaluating the impact the microbiome might have on brain disorders needs a much more detailed investigation in the future with spatial and also potentially time resolution.

## INTRODUCTION

In recent years, the impact of the human gut microbiota on brain function has come into focus, and the contribution of microbial communities to diseases such as Alzheimer’s disease (AD) has been intensively studied (e.g., reviewed in references 1, 2]. Several studies have attempted to unravel the existence of a disease-driving microbiome, but the results are still very inconclusive (summarized in reference 3, for a recent meta-analysis, see reference 4). Nevertheless, for some neurological diseases, such as AD and Parkinson’s disease (PD), there is evidence from preclinical models that at least the characteristic aggregation-prone peptides can be transferred from the gut to the brain. For example, injection of α-synuclein into the duodenal and pyloric muscularis layer of mice resulted in motor deficits and spread of the peptide in the brain (5).

In addition, transfer of fecal material from disease model mice or human patients to wild-type mice resulted in the expression of disease features (6). Additionally, at least some reports of probiotics administration describe beneficial effects in rodent models and less so in humans (summarized in reference 7). As the neurotoxic peptides typical for AD and PD have been shown to be bacteriotoxic, it is difficult to distinguish elicitor from bystander effects in the microbiota (8, 9). For example, a single intestinal passage of orally administered Aβ led to changes of the gut microbiota in mice; however, the microbiota of 5xFAD AD model mice seem to have somehow adapted to the chronic exposure (10). The use of germ-free mice may help to clarify this dilemma: germ-free AD model mice showed reduced Aβ plaque load, ameliorated inflammation, and increased Aβ degrading enzyme levels in comparison to conventionalized mice (11). Thus, reintroduction of single bacterial strains or restricted communities into germ-free mice may help to understand their contribution to pathogenic features.

The pre-dominant gut commensal *Bacteroides thetaiotaomicron* (hereafter abbreviated as *B. theta* or *B. t*.) showed anti-inflammatory properties in a mouse model of Crohn’s disease and was able to restore the neurochemical aberrant phenotype of enteric neurons and, thus, gut peristalsis after monocolonization of formerly germ-free C57BL/6 mice (12, 13). Here, we aimed to investigate the effect of *B. theta* re-introduction on brain features associated with AD in wild-type mice. Wild-type mice instead of AD model mice were used in the study to prevent potential fostered developmental changes due to the genetically evoked phenotype as it is known that the condition of being germ-free has impact on development *per se*. We chose to study three different brain regions that show different susceptibility to the neurodegenerative disease in human patients but also for pathological traits in mouse models: the hippocampus and the PFC are affected quite early in the pathogenesis, whereas cerebellar Aβ-deposits occur in the latest phase of the pathogenesis (14). Therefore, the cerebellum is thought to maintain its functionality until a late stage of the disease. Neuronal number, general anatomy, and presynaptic densities were examined in the three brain regions by immunohistochemistry. Furthermore, amyloid precursor protein (APP) processing was assessed by Western blotting, reactivity of the immune system was assessed by quantifying mRNA levels of specific receptors via qPCR, inflammasome markers via Western blotting, and kynurenine pathway was investigated via mass spectrometry.

## RESULTS

Germ-free male mice aged 4–6 weeks were gavaged with *B. theta* suspension and housed under incubator conditions for further 4 weeks (Fig. S1). Persistence of *B. theta* was confirmed by PCR using fecal samples at week 2, 3, and 4 post inoculation (15). At dissection, the left hemisphere of the mice was preserved for immunohistochemistry, while the right hemisphere was used for sub-region dissection. Hippocampus, PFC, and cerebellum were grinded under liquid nitrogen, and ¼ of the tissue was subjected to biochemical analyses (APP processing, qPCR of microglial receptor mRNA levels, expression of inflammasome components, and metabolic measures).

### Monocolonization by *B. theta* increases the number of presynaptic boutons in the CA1 region of the hippocampus

**Fig 1:**
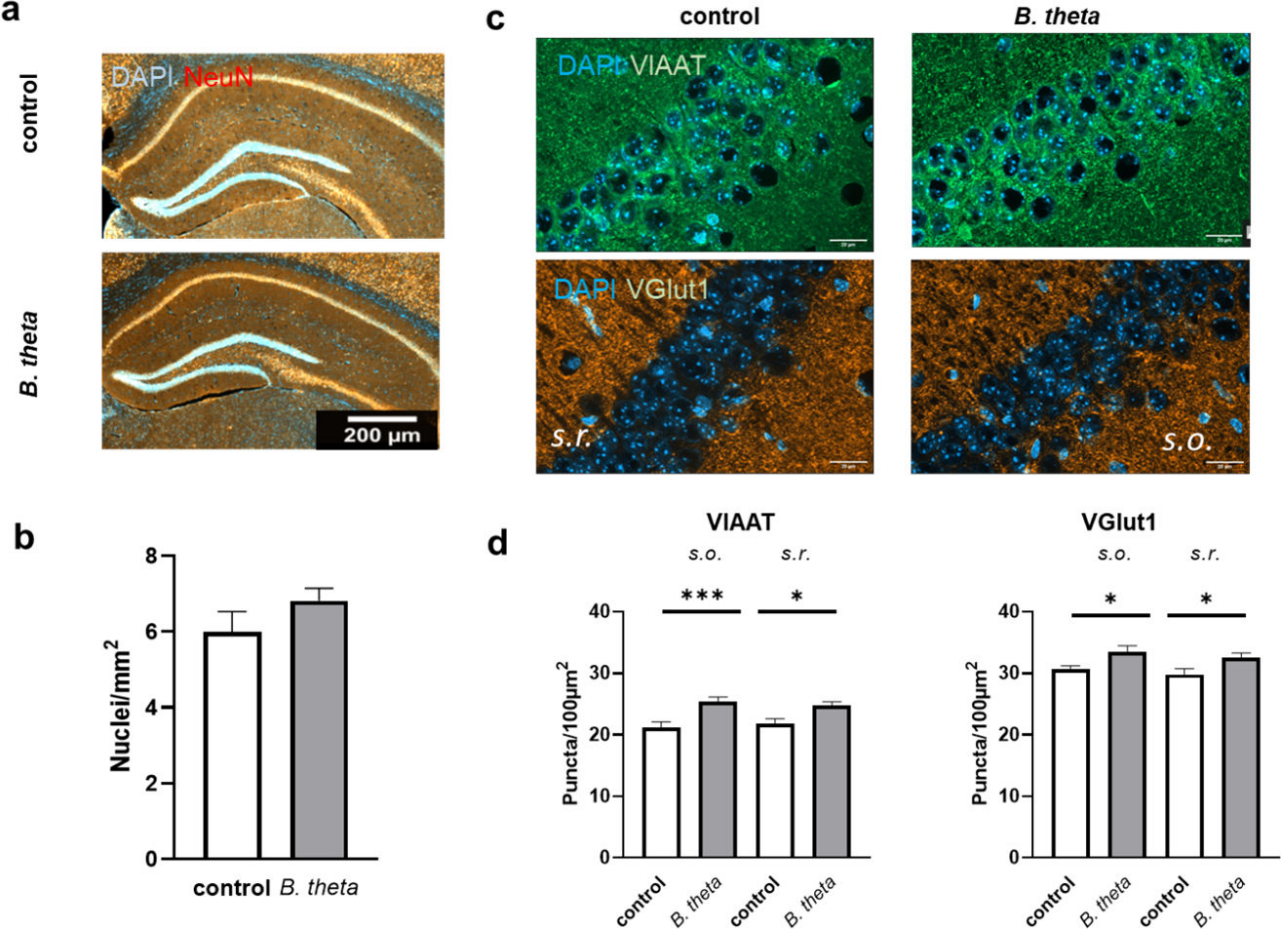
Neuron number and presynaptic density in hippocampal CA1 region of germ-free mice after *B. theta* monocolonization. (**a**) Brains were sliced and stained for nuclei (DAPI) and mature neurons (NeuN). Scale bar: 200 µm. (**b**) Nuclei were counted and number per mm^2^ calculated. (**c and d**) Additionally, counterstaining with VIAAT- or VGlut1-directed antibodies was performed, and synaptic puncta were counted for an area of 100 µm^2^ in both stratum oriens (s.o.) and stratum radiatum (s.r.). Data are presented as mean + SEM (*n* = 7 mice per group). Scale bar: 20 µm. Statistical significance was tested by Student’s *t*-test (**b**) or by one-way ANOVA with Fisher’s LSD post-test (**d**) (*, *P* < 0.05; ***, *P* < 0.001).

It is known that germ-free status affects several organ systems of the mouse, such as the gut, but also the brain. For example, the expression of synapse-related genes has been shown to be altered (summarized in reference 16). We, therefore, examined the number of neurons and the density of excitatory and inhibitory presynapses in the CA1 region of the hippocampus, a tissue related to episodic memory that is severely affected in AD (17). We observed no gross anatomical effects or changes in the number of NeuN-positive cells or nuclei in *B. theta*-treated mice compared to germ-free controls (Fig. 1a and b; *P* = 0.2166; 95% CI: −0.5518 to 2,198). However, the number of excitatory and inhibitory synaptic inputs was significantly increased for about 10%, as indicated by stainings of the vesicular inhibitory amino acid transporter (VIAAT) (Fig. 1c and d; *P* = 0.0010 and 0.0115; 95% CI −6,529 to −1,899 and −5,383 to −0.7524) and the vesicular Glu transporter type 1 (VGlut1) (Fig. 1c and d; *P* = 0.0312 and 0.0316; 95%CI −5,208 to −0.2684 and −5,202 to −0.2624; for a higher magnification to better identify individual puncta, see Fig. S2).

### Monocolonization with *B. theta* increases the number of synaptic puncta in the lamina molecularis of the PFC

**Fig 2:**
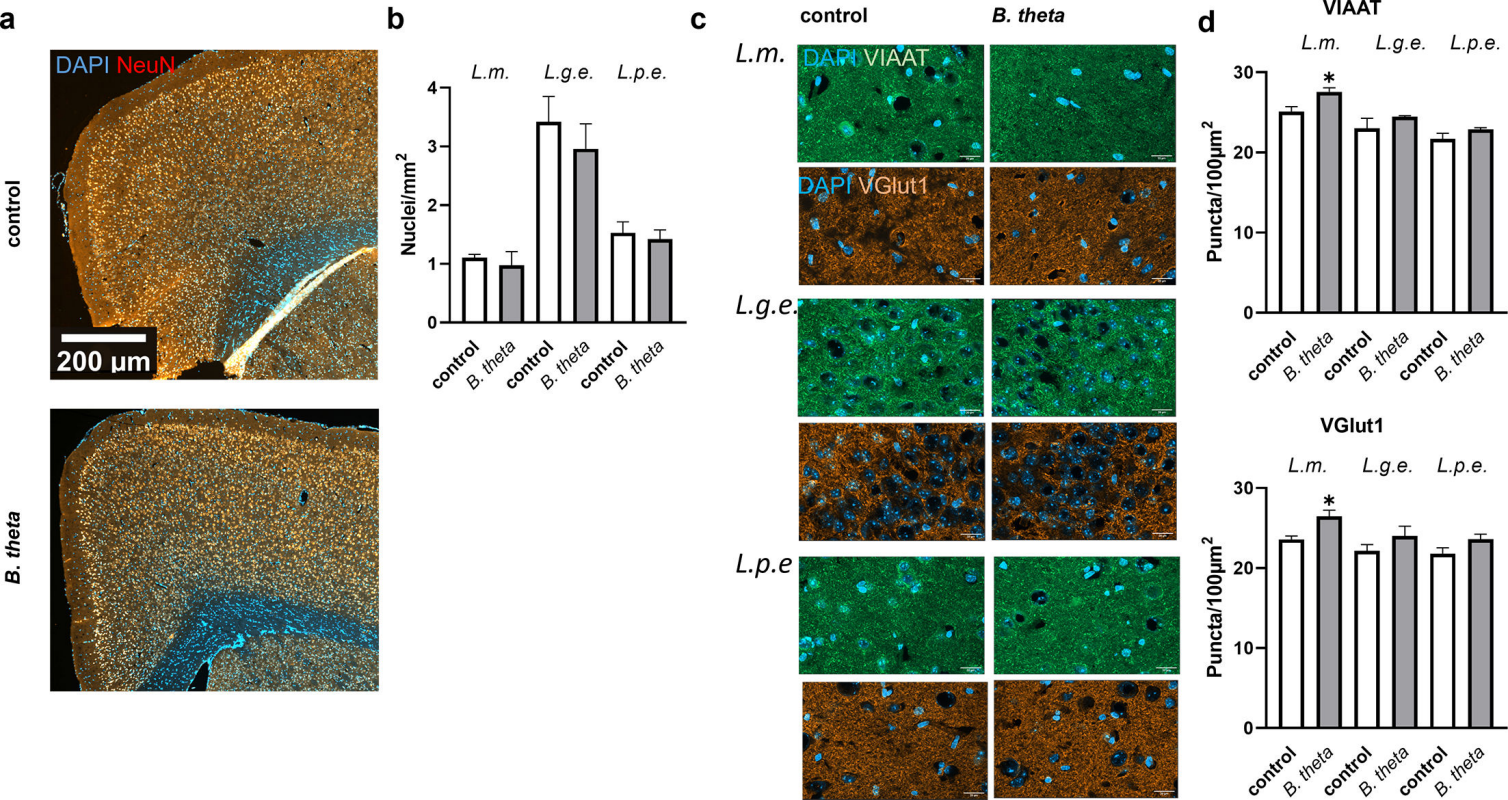
Number of neurons and structural features of the PFC after *B. theta* monocolonization of germ-free mice. (**a**) Brains were sectioned and stained for nuclei (DAPI) and mature neurons (NeuN). Scale bar: 200 µm. (**b**) Nuclei were counted, and the number per mm^2^ was calculated. (**c and d**) Counterstaining with VIAAT- or VGlut1-directed antibodies was performed and synaptic puncta were counted in an area of 100 µm^2^ in the lamina molecularis (*L.m*.), lamina granularis externa (*L.g.e*.), and lamina pyramidalis externa (*L.p.e*.). Data are expressed as mean + SEM (*n* = 3 mice per group). Scale bar: 200 µm. Statistical significance was tested by one-way ANOVA followed by Fisher’s LSD post-test (**d**) (*, *P* < 0.05).

The PFC is a heterogeneous cortical area associated with higher cognitive functions such as working memory or verbal abilities in humans. It has been shown to degenerate not only in AD but also in healthy aging (18). Defining this region in mice has been difficult and controversial, but recent studies are increasing our knowledge of its architecture and function in mice (19, 20). Similar to the hippocampus, no general effect on neuronal number could be observed in the PFC after reintroduction of *B. theta* into the mouse gut (Fig. 2a and b; *P* = 0.7516, 0.2733, and 0.7995, 95% CI −0.7466 to 1.007, −0.4149 to 1.339, and −0.7724 to 0.9814); however, a selective increase for synaptic puncta of 9% to 12% (both, VIAAT- and VGlut1-positive; *P* = 0.0257 and 0.0242; 95% CI −4.526 to −0.3502 and −5.330 to −0.4477) was assessed in the lamina molecularis*,* while other layers did not show such changes (Fig. 2c).

### Monocolonization by *B. theta* has no effect on cerebellar structure and neuron number

**Fig 3:**
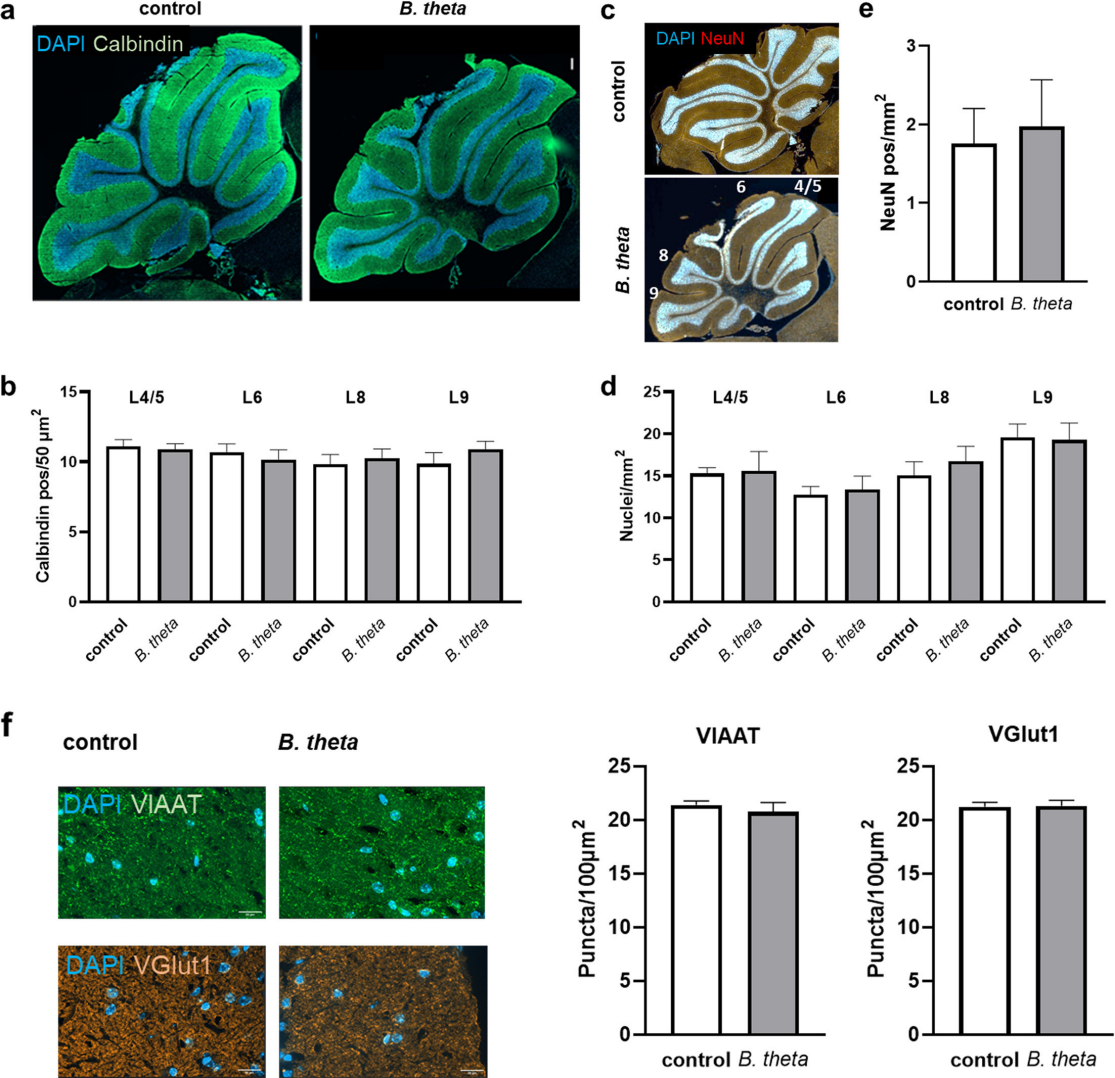
Cerebellar anatomy and neuron counts of germ-free mice colonized with *B. theta*. (**a**) Brains were sectioned and stained for nuclei (DAPI) and Purkinje cells (calbindin). Scale bar: 100 µm. (**b**) Calbindin-positive cells in the indicated lobes (see c) were counted and numbers calculated per 50 µm^2^. (**c**) Additionally, NeuN counterstaining was performed (white numbers indicate cerebellar lobes) and total neurons (**d**) and mature neurons (**e**) were counted per mm^2^. (**f**) Synaptic puncta were counted for an area of 100 µm^2^ after staining with VIAAT- or VGlut1-specific antibodies. Data are expressed as mean + SEM (*n* = 6 mice per group). Statistical significance was tested by Student’s *t*-test (**e and f**) or by one-way ANOVA with Fisher’s LSD post-test (**b and d**).

The cerebellum has long been thought to be spared from pathological changes until a very late stage of AD (14). Therefore, it has often been used as a kind of control tissue in AD (critically discussed in reference 21). When comparing germ-free control mice and *B. theta*-monocolonized animals, we did not observe any obvious anatomical changes, e.g., by calbindin staining, which labels pyramidal neurons (Fig. 3a). Furthermore, the number of calbindin-positive neurons in all investigated cerebellar lobes was not different between colonized and control mice (Fig. 3b).

Similarly, the number of total neurons (DAPI stain; Fig. 3a and d) as well as the number of mature neurons as indicated by their NeuN positivity (Fig. 3c and e) was unaffected. In addition, no change in VIAAT- or VGlut1-density was observed (Fig. 3f; *P* = 0.5234 and 0.8930; 95% CI −2.731 to 1.481 and −1.402 to 1.588).

### *B. theta* reintroduction into germ-free wild-type mice alters α-secretase activity

A hallmark of AD is abnormal processing of APP. This protein can be cleaved by various proteinases (for a review on non-canonical cleavage products, see reference 22), but two pathways are of particular relevance: the non-amyloidogenic pathway leads—via α-secretase activity—to a secreted ectodomain sAPPα. This molecule has been shown to be neuroprotective (reviewed in reference 23). Alternatively, β-secretase activity leads to the sAPPβ fragment, and after γ-secretase cleavage, neurotoxic Aβ peptides are released from respective C-terminal fragments (CTFs).

**Fig 4:**
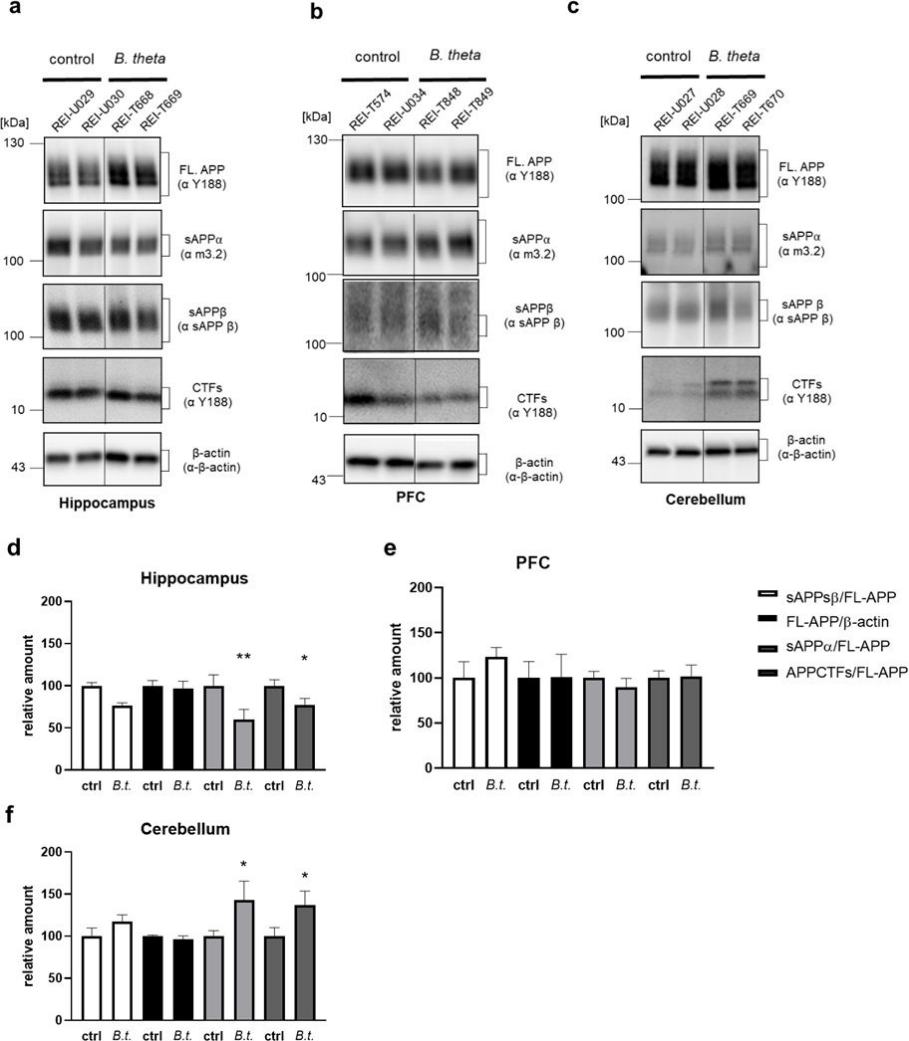
APP processing products in different brain regions after monocolonization of wild-type mice with *B. theta*. Brain sub-regions were minced under liquid nitrogen, and proteins were subjected to gradient SDS PAGE. Full-length APP (FL-APP), the secreted ectodomains sAPPα and sAPPβ, and the C-terminal fragments (CTFs) were detected by Western blotting using the respective primary antibodies (indicated in the blot images) in hippocampus (**a**), PFC (**b**), and cerebellum (**c**). The detection of β-actin was used as a loading control and for normalization. Two exemplary samples (labeled with animal identifiers, e.g., REIU029) are shown per group and tissue. (**d–f**) Densitometric analysis of protein bands was conducted and normalization performed using values for FL-APP or, in the case of FL-APP, for β-actin (*B. theta: B.t*.). Data are presented as mean + SEM (*n* = 10 mice per group). Statistical significance was tested by one-way ANOVA followed by Fisher’s LSD post-test (*, *P* < 0.05; **, *P* < 0.01).

The hippocampus showed a decrease of 40% and 25% in sAPPα and CTFs in animals inoculated with *B. theta* compared to control-treated germ-free mice (Fig. 4a and d; *P* = 0.0012 and 0.0427; 95% CI 16.50 to 63.94 and 0.8105 to 46.99), while full-length APP (FL-APP) was not affected. However, the PFC showed no effect on any of the quantified APP processing products (Fig. 4b and e; *P* = 0.6125, e.g., sAPPα). In the cerebellum, the opposite effect was observed compared to the hippocampus: sAPPα and CTFs were increased in *B. theta*-receiving mice to approximately 150% of germ-free mice (Fig. 4c and f; *P* = 0.0131 and 0.0250; 95% CI −76.48 to −9.300 and −68.47 to −4.735). Interestingly, the levels of sAPPβ, the product of β-secretase cleavage, remained unaffected in all tissue types tested (Fig. 4). This may indicate that, in our study, it was mainly the enzymatic activity of α-secretase that was regulated by the microbial commensal.

### Effect of *B. theta* monocolonization on immunological functions

**Fig 5:**
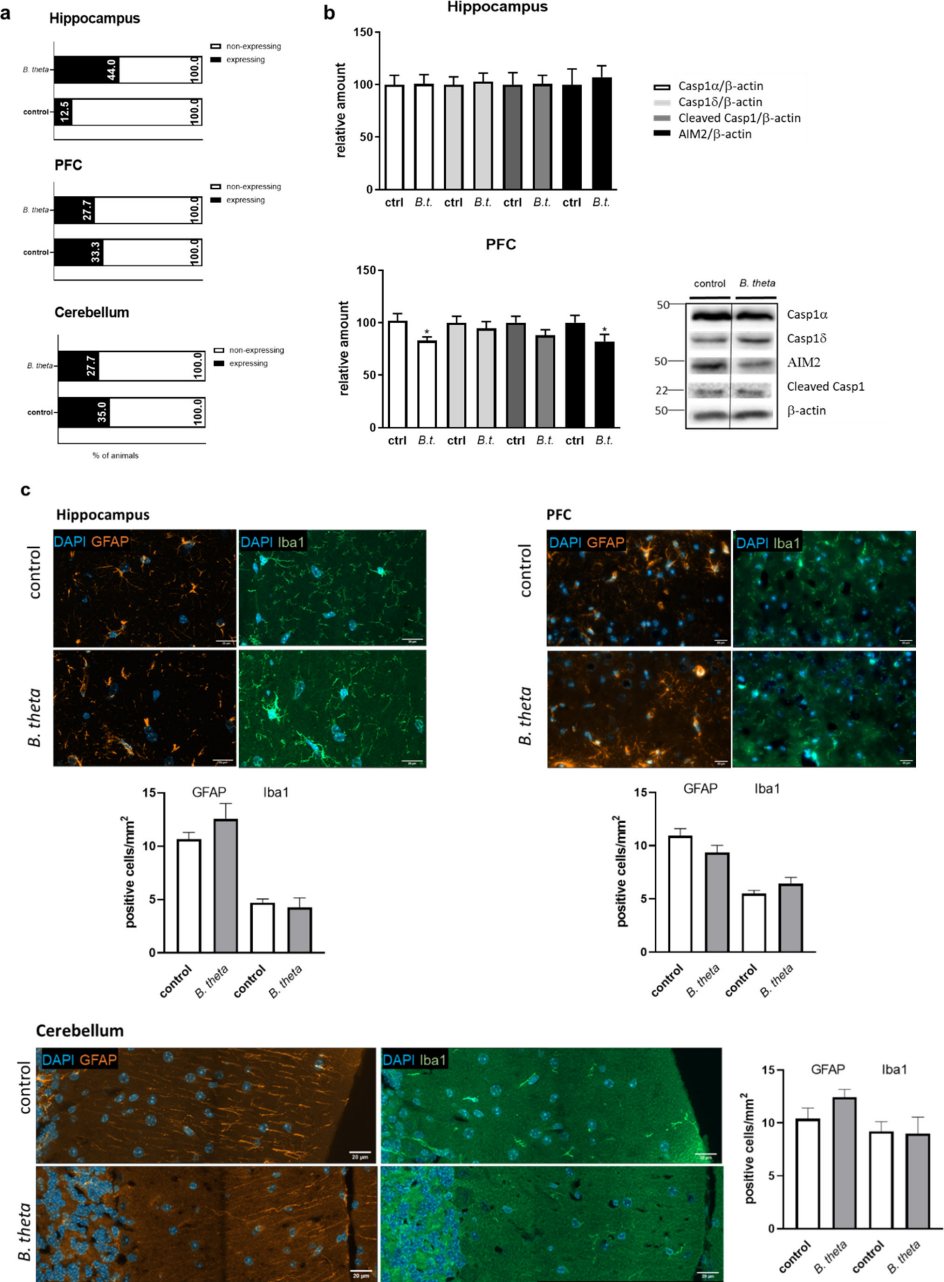
Effect of monocolonization of wild-type mice with *B. theta* on immune receptors, inflammasomal components, and immune cells of the brain. (**a**) RNA from the three respective brain regions was transcribed into cDNA, which was used for FPR1- and 2-specific qPCR. Detected receptor-characteristic DNA fragments are intron-free, and therefore, a strict control for genomic DNA contamination (-RT control) was performed (data not shown). All data failing this control were excluded from further analysis. Indicated are samples with detectable expression of either receptor in % of all included animals (*n* = 4–10 per group). (**b**) Tissue homogenates were analyzed by Western blotting by using an inflammasome antibody panel. Few protein-specific antibodies showed detectable signals, and cerebellar tissue did not show the respective proteins at all (*n* = 8–10 animals per group). (**c**) Quantitation of GFAP- and Iba1-positive cell numbers was performed using IHF images with DAPI counterstain (*n* = 3 animals for PFC, *n* = 6 for hippocampus and cerebellum). Scale bar: 20 µm. Data are presented as mean + SEM. Statistical significance was tested by one-way ANOVA with Fisher’s LSD post-test (*, *P* < 0.05).

Finally, as inflammatory processes are a major suspect in the pathogenesis of sporadic AD, the brain subregions of the mice were examined for the expression of immune receptors and inflammasome components after *B. theta* was introduced into the gastrointestinal tract. Formyl peptide receptors (FPR) are expressed on a wide variety of body cells and have been identified for their reactivity toward formyl peptides, typical characteristics of bacteria. However, they are also known to respond to host-derived protein fragments, and we have recently demonstrated that they are sensitive detectors of Aβ peptides and truncated Aβ variants (22). In general, FPRs are expressed in the rodent brain with a relatively high expression in brainstem but also in the PFC (24). We observed mostly limited and very divergent mRNA levels of both FPR1 and 2, while FPR3 was below detection level. This observation, probably due to the aseptic status of the animals, was further complicated by the structure of the genes, which lack introns in the respective amplified regions, making qPCR-based quantitation prone to error. We, therefore, decided to stratify the animals into expressers and non-expressers (meaning below detection level; sum of observations for FPR1 and 2, Fig. 5a) to gain insight into the effect of *B. theta* on this receptor class. While for PFC and cerebellum, the percentages of *B. theta* monocolonized mice with FPR expression did not diverge from those of germ-free control mice (about 30% in all cases), a higher proportion of animals displayed hippocampal expression of FPR1/2 after bacterial inoculation (Fig. 5a, binomial test observed [44%] vs expected [12.5%]: *P*<0.0001).

FPRs mainly detect extracellular ligands of potential bacterial origin and subsequently modulate the Nlrp3 inflammasome (25). The Aim2 inflammasome detects the presence of foreign DNA accumulating in the cytosol of cells but has also been implicated in self-reactivity (26). In germ-free mice, the expression of inflammasome components of both pathways appeared to be comparably low, as, for example, we were unable to detect Nlrp3 associated proteins in tissue lysates in general. Aim2 mRNA levels have been reported to be 6.2- and 18.2-fold higher than those of Nlrp3 and Nlrc4, at least in the developing mouse brain (27). We hypothesized that such differences would still be present in older animals, and thus, we could observe Aim2 and caspase 1α and δ in PFC and hippocampal tissue (Fig. 5b): while in hippocampus all components were comparable between germ-free and *B. theta*-colonized mice, the PFC showed a significant reduction in caspase 1 α and Aim2 itself in the presence of *B. theta,* while cleaved caspase1 showed a non-significant trend of decrease. The observed effects did not seem to correspond to mere amount of brain-resident immune cells, as both astrocytes and microglia (demonstrated by GFAP and Iba1 staining, Fig. 5c) were indistinguishable between control- and *B. theta*-treated mice in all three brain tissue specimen (e.g., *P* = 0.1551 and 0.7275 for GFAP and Iba1 in hippocampus).

The kynurenine pathway (KP) is critical for the supply of cellular energy in the form of nicotinamide adenine dinucleotide (NAD^+^). As immune responses are associated with increased energy requirements, the KP is also indicative of immune responses. In addition, kynurenine derivatives have been shown to be neuroactive, modulate neuroplasticity, and/or exert neurotoxicity. Thus, changes in the pathway have been investigated for correlation with AD and have even been discussed as biomarkers (28–30). We measured levels of tryptophan and kynurenine, the first product in tryptophan catabolism, in mouse brain tissue.

**Fig 6:**
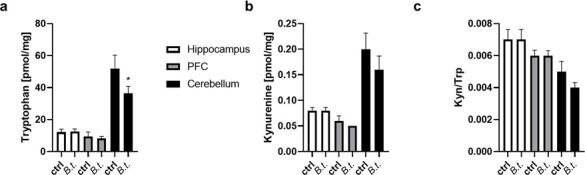
Tryptophan catabolism in brain regions of *B. theta*-inoculated mice. The respective brain sub-regions were subjected to mass spectrometry and tryptophan (**a**) and kynurenine (**b**) as well as tryptophan/kynurenine ratio (**c**) were quantified using the respective standards. Mean and SEM are given (*n* = 9–10 per group). Statistical significance was tested by one-way ANOVA followed by Fisher’s LSD post-test (*, *P* < 0.05).

While tryptophan levels were comparable in the hippocampus and PFC of control or *B. theta*-inoculated mice (Fig. 6a; *P* = 0.8304 and 0.9438), significantly reduced levels of about 70% of control were observed in the cerebellum of mice after recolonization with the bacterium (*P* = 0.0120; 95% CI 3.539 to 2.40). Kynurenine levels were not affected in the hippocampus and PFC (*P* = 0.6924 and >0.9999), and only a small, non-significant decrease was observed for cerebellum (Fig. 6b; *P* = 0.1176) similar to the findings for kynurenine/tryptophan ratio (Fig. 6c; e.g., *P* = 0.1655 for cerebellum).

**Fig 7:**
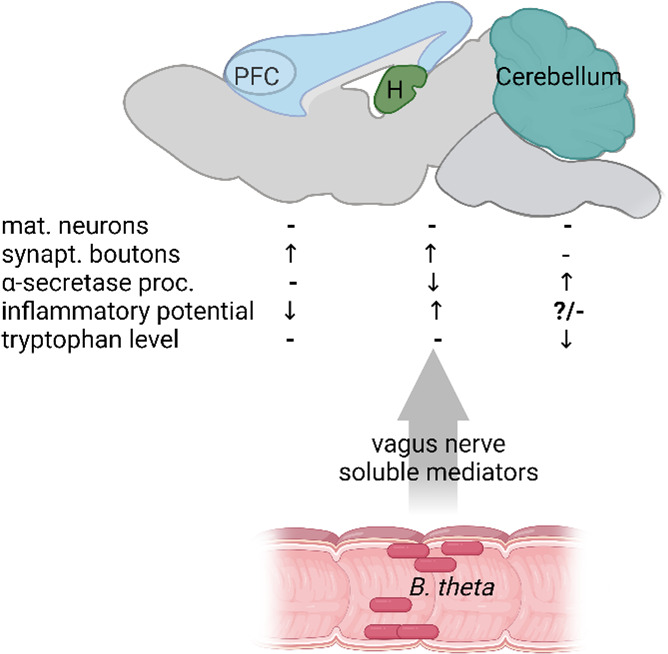
Schematic of observed CNS effects of *B. theta* monocolonization of the mouse gut. Three brain regions, hippocampus (H), PFC, and cerebellum, were investigated and showed a highly specific response pattern. Whether the effects are mediated by the vagus nerve or by soluble mediators such as lipopolysaccharides or short chain fatty acids cannot be deduced from our study and remains to be elucidated. The diagram was created by using BioRender (agreement number NQ28PM2GT8).

In sum, we provide evidence for a close interplay between the gut populated by bacteria and the brain. However, we must also conclude that within the brain, different areas respond differently to the resident organisms or their interaction with the host (see schematic in Fig. 7). This results in the modulation of pathways that have been linked to neurological disorders such as AD.

## DISCUSSION

Over the last decade, it has become clear that the microbiome plays a fundamental role in host health and disease (31, 32). While the impact of the microbial community *per se* has been documented in numerous studies, it is still difficult to disentangle the contribution of individual microbial organisms to the observed effects, as they form an immensely complex network based on competition, collaboration, and opportunistic traits. The difficulties arising from these mutual interactions may be reflected in the ongoing search for what might be called an AD microbiome. As a result, the field has moved toward gaining insight into causalities through manipulations such as fecal material transfer or recolonization of germ-free or antibiotics-treated mice, rather than solely analyzing individual microbial compositions. These studies mainly focus on functional outcomes such as the improvement of learning deficits in AD mouse models and on brain areas severely affected by the disease such as the hippocampus (e.g., see references 11, 33, 34). Here, we analyzed different molecular players known to be involved in AD in three different brain areas of wild-type mice after introducing a single bacterium common to humans and mice to reduce complexity and better understand whether a single microbial organism affects the brain *per se* in a global way or whether different brain sub-regions react specifically.

### Regulation of neurons by monocolonization with *B. theta*

The number of cell nuclei in all three brain regions, PFC, hippocampus, and cerebellum, was not affected by inoculation of mice with *B. theta*. However, the density of glutamatergic and GABA-ergic presynaptic terminals was increased in hippocampus and in the lamina molecularis of the PFC. Synaptic loss is one of the hallmarks of AD, and glutamatergic hypoactivity has been shown, for example, in Aβ-based AD mouse models (35). Interestingly, the rTg(TauP301L)4510 AD model showed the opposite, namely, hyperexcitability of hippocampal neurons and increased extracellular glutamate (36). In young APPPS1 mice (1–3 months), increased GABAergic synaptic output of hippocampal PV+ interneurons was demonstrated (37). In rats, intra-hippocampal microinjection of a GABAB receptor antagonist reversed Aβ-induced learning, memory, and cognitive impairments (38). The interpretation of the finding that both glutamatergic and GABA-ergic terminals showed increased staining after *B. theta* inoculation is, therefore, complex. In particular, we cannot say whether this is due to increased insertion of the transporters into synaptic vesicles or to an increased number of synapses. Interestingly, germ-free mice are characterized by reduced neuronal and vagal afferent innervations within the gut. This could be restored by conventionalization by *B. theta* via an increase of nitric oxide synthase (NOS)-expressing inhibitory neurons and excitatory cholinergic marker choline acetyltransferase (ChAT)-expressing neurons in the colon, which parallels our findings for simultaneous increase in specific brain regions (13).

The cerebellum, the third region we studied, has long been thought to be resistant to neurodegeneration, perhaps because the motor functions of patients and mouse models are largely preserved, falsely suggesting that this region of the brain is unaffected. Only in the last few years, reports have been published showing that the cerebellum is not completely exempt from pathological changes. For example, the number of Purkinje cells was unchanged, as was Tau immunoreactivity in post-mortem human brain tissue microarrays from AD patients, but the number of vessels was increased and Iba1 expression was strongly induced (39). A meta-analysis of 54 studies showed cerebellar atrophy in AD, which is also distinct from age-related gray matter loss (40, 41). In the cerebellum, we did not observe any microbe-induced changes in presynaptic bouton density, which may indicate that this brain region has a higher homeostatic capacity and does not seem to respond easily to certain exogenous or endogenous signals.

### *B. theta*-driven effects on APP processing

Mice do not develop disease-typical Aβ deposits *per se* because of a different primary sequence in the peptide stretch that affects the propensity of the beta-strand to fold. However, they have the same secretases capable of cleaving APP. We, therefore, analyzed the amount of APP cleavage products corresponding to either β- or α-secretase: while in the PFC, both pathways seemed to be unaffected and β-cleavage was not affected in any tissue, the α-cleavage pathway was decreased in the hippocampal samples, whereas *B. theta* monocolonization led to an increase of the corresponding pathway in the cerebellum. This is noteworthy as increased α-secretase activity has been shown to be protective against AD and a decrease in its functionality has been linked to cognitive decline in the elderly (42, 43). Intense immunoreactivity for ADAM10 (A disintegrin and metalloproteinase 10) has been found in adult mouse brain not only in the hippocampus (stratum pyramidale of CA2 and 3) but also in the Purkinje and granular layer of the cerebellum (44). Thus, the major neuronal α-secretase ADAM10 (45, 46) is present in both brain areas but appears to be differentially regulated by the presence of *B. theta*. Different levels of ADAM10 regulation have been identified, ranging from transcription, translation, protein binding partners such as tetraspanins or interaction with lipids (47). As little is known about the potential factors in cerebellum, and ADAM10 is not only expressed in neurons but also in other brain cells, it remains enigmatic how this differential regulation occurs (48, 49).

### Neuroimmunity affected by *B. theta* monocolonization

The intensity and duration of the immune response within the brain have been strongly correlated with either protective effects or deleterious contribution to AD pathogenesis (50, 51). For example, both astrocytes and microglia in the brain respond to the neurotoxic Aβ peptides; however, astrocytosis or gliosis was not observed in any of the brain regions examined as a consequence of *B. theta* monocolonization. Germ-free mice have contributed to the understanding that the gut microbiota contributes profoundly to the host’s immunity, such as maturation of immune cells (52, 53). Thus, it was interesting to observe localization-specific responses when analyzing FPR1 and 2 expression and inflammasome components. FPR receptors represent a kind of cross-road between AD pathology and host reaction to microbial compounds. They have been described to bind not only to formyl-decorated peptides, indicating the presence of bacteria, but also to truncated Aβ peptides (54). Therefore, inducing their expression (in hippocampus) could also increase sensitivity to Aβ-driven immune responses, which could further lead to overstimulated conditions and, thus, progression of pathogenesis. Bacterial compounds can activate—via transmembrane or cytosolic pattern recognition receptors—the inflammasome, which has been shown to be integral in neuroinflammation. Nlrp3, for example, has been shown to be involved in Aβ and Tau-driven neurotoxic events in animal models of the disease (55). Only within the PFC were changes of inflammasome components observed with *B. theta* inoculation, with a significant reduction in Aim2 and caspase 1. Most recently, it has been shown that conditional knock-out of Aim2 in AD model mice was able to restore cognitive function as by the Morris water maze test, while Aim2 overexpression reduced neuronal complexity and dendritic spine intensity in the hippocampus (56). The PFC was not examined in this publication, but a beneficial effect of reduced Aim2 on AD pathology can be inferred.

### Changes in tryptophan metabolism induced by *B. theta* monocolonization

Tryptophan metabolism has been implicated in AD pathomechanisms in several ways: some metabolites appear to exert protective effects (e.g., 3-hydroxyanthranilate inhibiting Aβ oligomerization), while others such as quinolinic acid are neurotoxic (reviewed in reference 57). In mice monocolonized with *B. theta*, neither kynurenine nor tryptophan/kynurenine ratio was affected in any brain region. Only tryptophan was found to be reduced in the cerebellum when levels were compared with those in germ-free mice. Tryptophan is converted to kynurenine by indole amine 2,3-dioxygenase (IDO), an enzyme that responds to inflammatory cytokines and is thought to be involved in CNS disorders (58). As kynurenine levels were not found to be elevated, an increase in this catalytic step of the kynurenine pathway does not seem to be the underlying cause. This is also consistent with our findings that none of the tested components of the brain immune system were affected in the cerebellum. Therefore, a reduced availability of dietary tryptophan could be a possible explanation. About 5% of dietary tryptophan can be catabolized to indole derivatives by the gut bacteria and *B. theta* is known to produce indole-3-propionic acid, which has anti-inflammatory potential in the gut (59, 60). Why the cerebellum, in particular, shows reduced levels while hippocampus and PFC show unchanged levels cannot be resolved by this study. There are few studies on regional differences in tryptophan levels in the brain. For example, an early study on rat brain slices suggests that the cerebellum has an uptake rate comparable to that of the hippocampus or cerebral cortex (61). This would not account for our observations.

### Conclusion

To our knowledge, this is the first time of describing how colonization of germ-free mice with a single bacterial species affects different brain regions emphasizing AD pathology. This adds a layer of complexity to the debate about microbial influence on CNS health and disease.

Nevertheless, more data are needed in the future as our study entailed some limitations such as investigating a single post-colonization time point, demonstrating changes for selected immune targets only, and lack of behavioral anchoring (by this showing functional relevance). Moreover, potential sex-dependent effects would also need to be addressed in future investigations as for this study, only male mice were included and a mutual influence of microbiome and sex has been found.

## MATERIALS AND METHODS

### Animals

Male mice (C57Bl6/J, *n* = 10 per group) were obtained from germ-free housing (CTH, JGU Mainz) at the age of 4 to 6 weeks. They were given 200 µL of a *B. theta* suspension (see below) or pure medium (control) by gavage and then maintained in the germ-free housing for 4 weeks. From week 2, colonization was assessed by feces-based PCR (15).

### Bacteria

*B. theta* was obtained from the DSMZ (Deutsche Sammlung von Mikroorganismen und Zellkulturen GmbH; catalog number DSM2079) and stored at −80°C as a glycerol-containing stock. Prior to gavage, the bacteria were cultured in brain heart infusion broth medium (Sigma-Aldrich) and incubated at 37°C with continuous shaking (250 U/min) until they reached an OD_600_ of 0.6 (about 48 h).

### Detection of *B. theta* in fecal samples

Genomic DNA was extracted from cage-wise collected feces (Nucleo Spin Soil kit, Macherey-Nagel) and subjected to PCR using a universal primer pair to detect 16S-rDNA and the following specific primer pair: BTH-F: TGGAGTTTTACTTGAATG, BTH-R: CCCATTGTAAAAGGGC (62). *B. theta* colonization had already established stably 2 weeks after administration as reported previously (15).

### Dissection

Animals were anesthetized at the age of 8–10 weeks (mean age *B.t*.: 8.9 weeks, ctrl: 9.1 weeks, *P* = 0.62) with isoflurane and decapitated. The brain was removed without the olfactory bulbs and immediately shock frozen in liquid nitrogen (right hemisphere) or fixed (see below, left hemisphere).

### Immunohistochemistry

After brain removal, the left hemispheres were fixed in 4% paraformaldehyde at room temperature for 24 h and then immersed in 30% sucrose in PBS at 4°C for 48 h. Specimen were frozen in liquid nitrogen and stored at −80°C. The hindbrain was separated from the rest of the brain and both frozen in an OTC embedding medium (Roth KMA-0100-00A). Cryosections of 8 µm thickness were prepared and the coronal PFC and hippocampal samples and the sagittal slices of the cerebellum placed on microscope slides and fixed in ROTIHistofix 4% (Roth P087.5) for 8 min. The tissue was washed three times with ice-cold 1× PBS-T, followed by an antigen retrieval with 10 mM sodium citrate/0.05% Tween-20 (pH 6) at 90°C for 20 min. Slides were cooled for 20 min. After three washes with 1× PBS-T, a blocking solution (1× PBS/0.2% Triton/5% BSA/5% HS) was applied for 1 h at RT. The primary antibodies (Calbindin 1:200 Abcam ab108404; GFAP 1:400 Synaptic Systems 173011; Iba1 1:300 Wako 019-19741; NeuN 1:500 Sigma-Aldrich MAB377; VIAAT 1:300 Synaptic Systems 131004; VGlut1 1:300 Synaptic Systems 135304) diluted in 1× PBS/5% BSA/5% HS were applied to the tissue and incubated for 12 h at 4°C. After three washes in 1× PBS-T, the tissue was incubated for 1 h in Alexa-Fluor-conjugated antibodies diluted 1:300 in 1× PBS/5% BSA/5% HS. Staining with DAPI (5 µg/mL in 1× PBS; Sigma Aldrich D9564) for 20 min at RT was performed between two rounds of three 1× PBS-T washes. The tissue was rinsed with MilliQ water and sealed with Mowiol and a cover slip. Fluorescence images were taken using an Axio Observer Z.1 Microscope (Zeiss). Brains were imaged at 40× magnification using tiles. Z-stack images of presynaptic boutons were acquired at 63× magnification using Plan Apo 20×/0.8 DICII objectives (Zeiss) and oil immersion. ImageJ software was used for analysis (63).

For each animal and brain region, multiple IHC measurements were collected and averaged to obtain a single mean value per animal, which served as the basis for group-level statistics. The IHC analyses were performed on ≥2 sections per animal and region, with ≥3 ROIs per section. Only for NeuN density, 1 ROI per animal was analyzed. Images were auto-thresholded using the default settings to generate binary images. From these, multiple randomly selected 10 × 10 µm ROIs were extracted, and the “particle analysis” tool (0.1–∞ µm²) was applied to determine the number of particles within each ROI.

The original images were partly rotated or mirrored to align their orientations. Furthermore, black boxes were added to highlight labels and to cover imaging software scale bars (e.g., Fig. 3a and c).

### Tissue preparation for RNA and protein analysis

Brain subregions were dissected from frozen material on cooled metal plates. Hippocampus, cerebellum, and PFC were then minced under liquid nitrogen and divided into proportions used for subsequent RNA preparation, Western blotting, and metabolite analysis. Grinded tissue material was weighed to normalize the amount within each methodological approach.

### Western blotting

Minced brain tissue was dissolved in 1× SDS loading buffer including DTT (volumes adjusted to the sample weight). Samples were boiled at 95°C for 5 min to detect APP processing products. Proteins were separated by SDS gel electrophoresis on 4%–15% Tris-glycine gradient gels, and Western blot detection was performed using anti-CT APP α-Y188 (1:5,000, rabbit monoclonal, Abcam) to detect APP C-terminal fragments as well as full-length APP. The antibody α-m3.2 (1:200, mouse monoclonal, kind gift from Prof. Paul Matthews) was used to detect sAPPα and anti-sAPPβ (1:1,000, rabbit polyclonal, IBL) to visualize sAPPβ. Anti-β-actin antibody (1:1,000, mouse monoclonal, Sigma) was used to provide a loading control signal. Signals were quantified by measuring the integrated density using ImageJ.

Inflammasome components were detected by Western blot as previously described (64). The primary antibodies were as follows: caspase-1 (E2Z1C; 24232), cleaved caspase-1 (Asp296) (E2G2I; 89332), and AIM2 (D5X7K; 12948) (all 1:1,000, Cell Signaling). As a loading control, β-actin was detected (A2066; Sigma-Aldrich). Blots were incubated with the secondary antibody anti-rabbit IgG HRP-linked antibody (7074; Cell Signaling).

### RNA preparation and qPCR

Total RNA was isolated using the Analytik Jena innuPREP RNA kit (845-KS-2040050) according to the manufacturer’s protocol. cDNA was synthesized using 30 ng total RNA per sample with Superscript II Reverse Transcriptase (ThermoFisher Scientific, 100004925), deoxynucleoside triphosphates and SMART, CDS(smart) primers (Sigma-Aldrich) with the conditions as follows: 42°C for 60 min, 55°C for 15 min, and 65°C for 10 min.

qPCRs were performed using 0.5 µL of cDNA as well as forward and reverse primers and Biozym Blue S’ Green qPCR kit (331416) as duplicates in 20 μL total reaction volume according to the MIQE guidelines (65). qPCR conditions were as follows: 95°C for 3 min, 35 cycles at 95°C for 5 s, 68°C for 10 s followed by a final extension of 66°C for 15 s. Representative samples of all qPCR products were assessed for their quality by gel electrophoresis and sequencing. Absolute quantification of DNA copies was calculated from specific standard curves (dilution series of a sequenced and purified PCR product in 2 ng/μL yeast tRNA [Sigma-Aldrich]). In addition, relative quantifications were performed for a house keeping gene using mouse Gapdh. All PCRs were performed in a TOPical T-Gradient thermocycler (Biometra).

Primers were as shown in Table 1.

### Mass spectrometry and HPLC

**TABLE 1:** Oligonucleotides used for qPCR analysis

Accession no.	Gene	Official full name	Oligonucleotide sequence
qPCR primers			
NM_013521.2	mFpr1	*Mus musculus* formyl peptide receptor 1 (Fpr1), mRNA	5′-GGGCTCGTGATCTGGGTGGCT-3′ 5′-GCGGTCCAGTGCAATGAGGGC-3′
NM_008039	mFpr2	*Mus musculus* formyl peptide receptor 2 (Fpr2), mRNA	5′-CCGGATGCCACACACTGTCACCA-3′ 5′-TGCAGCGGTCCAAGGCAATGAGA-3′
NM_001289726	mGapdh	*Mus musculus* glyceraldehyde-3-phosphate dehydrogenase mRNA	5′-TGAACGGATTTGGCCGTATTGG-3′ 5′-TGCCGTTGAATTTGCCGTGAG-3′
Reverse transcription primers			
		CDS (Smart)	5′-AGCAGTGGTAACAACGCAGAGTACTTTTTTTTTTTTTTTTTTTTTTTTTTTTTTVN
		SMART II	5′-AAGCAGTGGTAACAACGCAGAGTACGCGGG

A stable heavy isotope labeled internal standard solution of tryptophan was used at a concentration of 1 mg/mL (^13^C_11_^15^N_2_-Trp, Toronto Research Chemicals, T202406). Calibration solutions for kynurenine (Kyn; Sigma Aldrich, K3750) and tryptophan (Trp; Sigma Aldrich, T0254) were prepared as described in a concentration rage of 50–6,000 nM for Trp and 3–300 nM Kyn, respectively (66). Ascorbic acid (Carl Roth, 6288.3) was dissolved in double-distilled water to give a 2% (wt/vol) solution. For Trp, a 1.000 mmol/L stock solution was prepared in 50% methanol (Merck, 1.06035.1000), 48.9% water, 1% ascorbic acid solution, and 0.1% formic acid (Fisher Chemical, T-33362). A 1.000 mmol/L stock solution of Kyn was prepared in 1% ascorbic acid solution and 0.1% formic acid in methanol.

Brain tissue was prepared according to reference 66. Briefly, 50 µL of acidified mobile phase and 10 µL of internal standard mixture were added to grinded tissue. For calibration standards, 50 µL of calibrator solution was mixed with 10 µL of internal standard. Then 200 µL of chilled methanol (−20°C) were added. Samples were homogenized twice for 10 s on ice using a supersonic homogenizer with a 20 s break between each round. Proteins were precipitated by incubation for 30 min at −20°C. Samples were cleared by centrifugation at 15,000 rcf at 4°C for 10 min. Two hundred microliters of supernatants was transferred to fresh reaction tubes and evaporated to dryness under a gentle nitrogen stream. Residues were reconstituted in 100 µL of acidified mobile phase, incubated for 10 min at 21°C and heavy shaking, and subsequently vortexed for 20 s. Samples were again cleared by centrifugation at 15,000 rcf at 4°C for 10 min. The supernatants were transferred to vials and measured.

The analysis was carried out on an Agilent 1290 HPLC, consisting of a binary pump (G4220A), a column oven (TCC G1316C), an autosampler (G4220A), and a thermostat (G1330B) (Agilent Technologies, Waldkirch, Germany), coupled with a QTrap 5500 tandem mass spectrometer (AB Sciex, Darmstadt, Germany). A reversed phase column (Xterra MS C18, 2.5 µm, 2.1 × 50 mm; Waters, Eschborn, Germany) was used for separation with a suitable guard column at 20°C. The mobile phases consisted of 0.1% acetic acid in water (A) and acetonitrile (B) at a flow rate of 400 µL/min. The gradient program was as follows: 0% B at 0 min, raised to 80% over 2 min, and held for 1 min, then %B was reduced to starting conditions over 2 min with a 1 min reconditioning step. An injection volume of 1 µL was used for Trp measurement and 10 µL for Kyn measurement. An electrospray source (ESI), operated in positive mode, was used for ionization. The source parameters were as follows: ion spray voltage 4,500 V, temperature 450°C, nebulizer gas 40 psi, heater gas 45 psi, curtain gas 25 psi. The measurement was carried out in the multiple reaction monitoring (MRM) mode. For each analyte, two mass transitions (Q1→Q3, *m/z*) were measured, first (marked with an asterisk) for quantification and second as confirmatory signal. For the isotopically marked standard (Trp-^13^C_11_-^15^N_2_), only one mass transition was used: Kyn 209.1→94.0*, 209.1→146.1; Trp 205.2→118.2*, 205.2→146.0; Trp-^13^C_11_-^15^N_2_ 218.2→156.2*. Gained amounts of either metabolite were normalized to tissue weight.

## Data Availability

The data for quantification of Kyn and Trp and metadata reported in this paper are available via MetaboLights (https://www.ebi.ac.uk/metabolights/MTBLS12896 study identifier MTBLS12896 [67]). Data for Western blots and regarding qPCR (e.g., efficiency calculations) are available via doi 10.5281/zenodo.17182756. All microscopic images from mouse brain are available on https://archive.nfdi4plants.org/records/rwpke-xgd54; doi: https://doi.org/10.60534/rwpke-xgd54.

## SUPPLEMENTAL MATERIAL

Figures S1 and S2 spectrum.00744-25-s0001.docx Experimental schematic and staining of CA1 region.

ASM does not own the copyrights to Supplemental Material that may be linked to, or accessed through, an article. The authors have granted ASM a non-exclusive, world-wide license to publish the Supplemental Material files. Please contact the corresponding author directly for reuse.
