# Supplementary material for: Monocolonization with Bacteroides thetaiotaomicron exerts region-specific effects on Alzheimer’s disease-related traits in the murine brain
Source: Microbiol Spectr. 2026 Jan 20;14(3):e00744-25. doi: 10.1128/spectrum.00744-25 (PMC12955396; doi:10.1128/spectrum.00744-25)
Supplement: Figures S1 and S2 — Experimental schematic and staining of CA1 region. [file spectrum.00744-25-s0001.docx]

Monocolonization with *Bacteroides thetaiotaomicron* exerts region-specific effects on Alzheimer’s disease-related traits in the murine brain

Vu Thu Thuy Nguyen*^1^, Svenja König*^2^, Henning Formes*^3^, Zukaa Al Taleb^4^, Florian Steinert^2^, Bernd Bufe^4^, Simone Eggert^2,5^, Simone Stegmüller^6^, Yannik Schermer^6^, Elke Richling^6^, Stefan Kins^2^, Christoph Reinhardt^3^, Kristina Endres^1^

Supplementary Figures


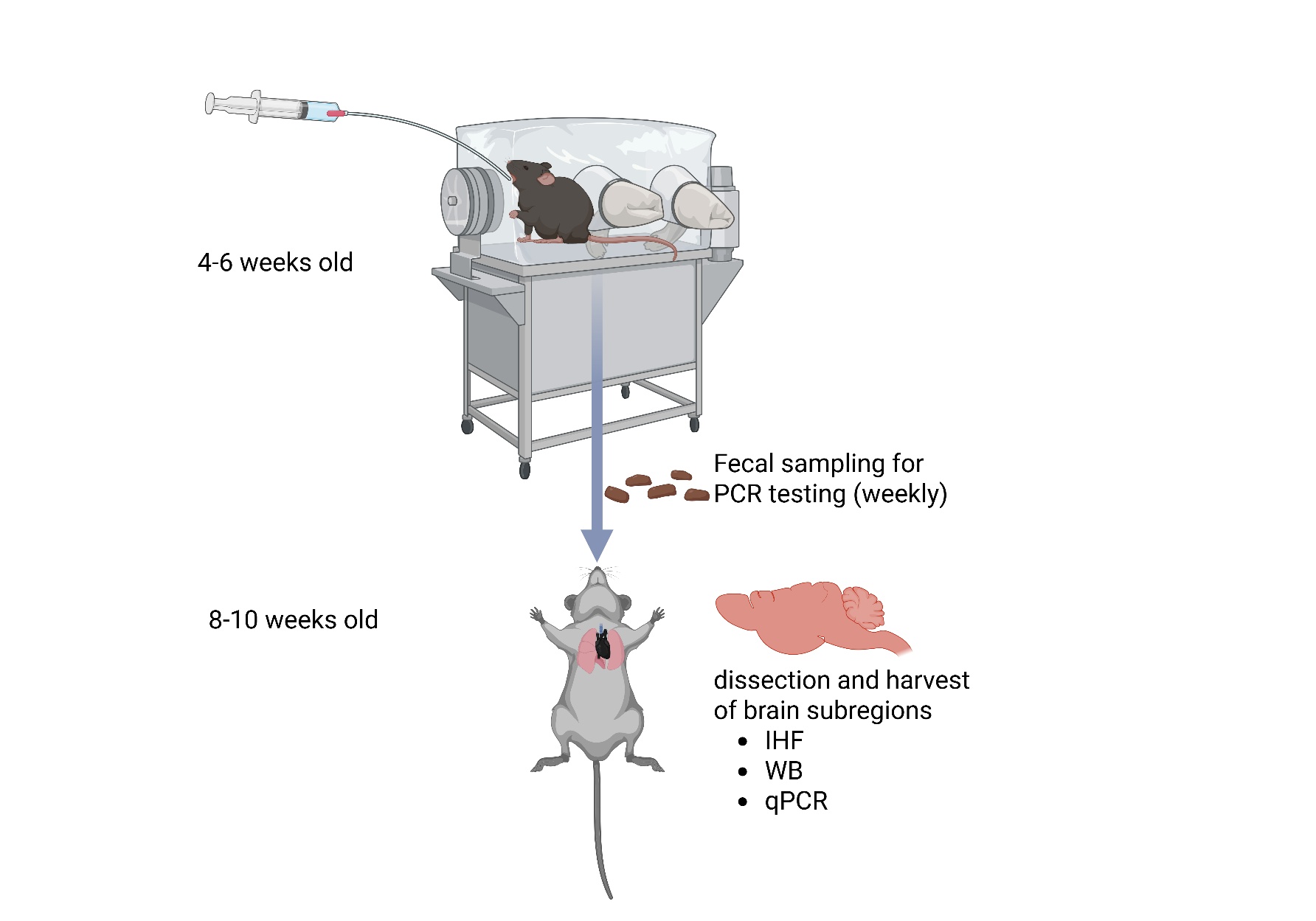


Supplementary Figure 1. Schematic of inoculation and experimental procedure. The image was created by using BioRender (agreement number DC28PLPNVZ).


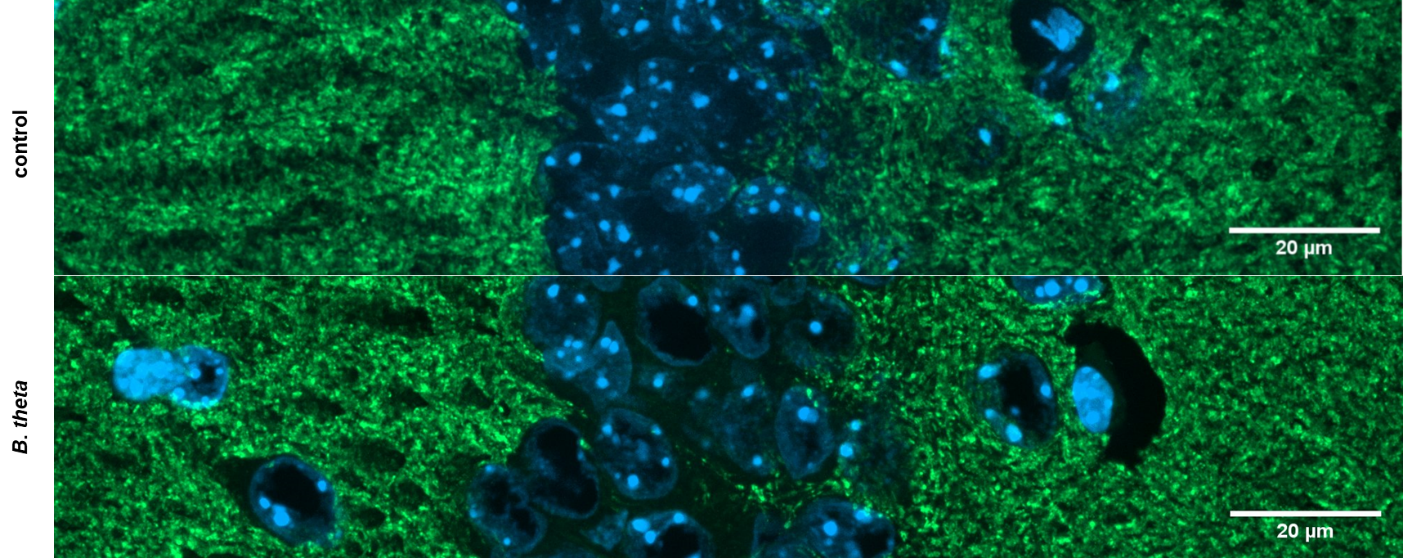


Supplementary Figure 2. Staining of hippocampal CA1 region of germ-free mice after *B. theta* conventionalization with VIAAT-specific antibody (green). DAPI staining (blue) was used to visualize cell nuclei.
